# Compound Raman microscopy for rapid diagnosis and antimicrobial susceptibility testing of pathogenic bacteria in urine

**DOI:** 10.3389/fmicb.2022.874966

**Published:** 2022-08-24

**Authors:** Weifeng Zhang, Hongyi Sun, Shipei He, Xun Chen, Lin Yao, Liqun Zhou, Yi Wang, Pu Wang, Weili Hong

**Affiliations:** ^1^Institute of Medical Photonics, Beijing Advanced Innovation Center for Biomedical Engineering, School of Biological Science and Medical Engineering, Beihang University, Beijing, China; ^2^School of Engineering Medicine, Beihang University, Beijing, China; ^3^Department of Urology, Peking University First Hospital, Beijing, China; ^4^Department of Clinical Laboratory, China Rehabilitation Research Center, Capital Medical University, Beijing, China

**Keywords:** antimicrobial susceptibility testing, bacterial identification, compound Raman microscopy, stimulated Raman scattering, Raman spectroscopy

## Abstract

Rapid identification and antimicrobial susceptibility testing (AST) of bacteria are key interventions to curb the spread and emergence of antimicrobial resistance. The current gold standard identification and AST methods provide comprehensive diagnostic information but often take 3 to 5 days. Here, a compound Raman microscopy (CRM), which integrates Raman spectroscopy and stimulated Raman scattering microscopy in one system, is presented and demonstrated for rapid identification and AST of pathogens in urine. We generated an extensive bacterial Raman spectral dataset and applied deep learning to identify common clinical bacterial pathogens. In addition, we employed stimulated Raman scattering microscopy to quantify bacterial metabolic activity to determine their antimicrobial susceptibility. For proof-of-concept, we demonstrated an integrated assay to diagnose urinary tract infection pathogens, *S. aureus* and *E. coli*. Notably, the CRM system has the unique ability to provide Gram-staining classification and AST results within ~3 h directly from urine samples and shows great potential for clinical applications.

## Introduction

Antimicrobial resistance (AMR) can cause severe or even life-threatening complications, such as sepsis and urinary tract infection (UTI; Davenport et al., 2017; Syal et al., 2017; Florio et al., 2018). Traditional bacterial identification and antimicrobial susceptibility testing (AST) methods are usually based on culture, typically taking several days (Dietvorst et al., 2020; van Belkum et al., 2020). This slow process delays the appropriate medical decision and prompts clinicians to use antibiotics based on their experience or broad-spectrum antibiotics, leading to an abuse of antibiotics. Therefore, accurate and timely identification and AST of microorganisms is essential to help clinicians initiate the most effective therapy.

Many emerging techniques have been developed to achieve faster identification or AST (Syal et al., 2016; Li et al., 2017; Schoepp Nathan et al., 2017; Hilton et al., 2020; Klein and Hultgren, 2020; Pragadeeshwara Rao et al., 2020; Chen and Hong, 2021). For example, molecular diagnostic methods based on the detection of specific genes, such as polymerase chain reaction (PCR; Dietvorst et al., 2020), clustered regularly interspaced short palindromic repeats (CRISPR; Chen et al., 2020), whole-genome sequencing (Flenker et al., 2017), and DNA microarray (Schoepp Nathan et al., 2017), provide faster identification results. However, molecular methods are not generally applicable to all bacterial species or mechanisms. Moreover, they lack sensitivity when detecting all organisms present in various microbial cultures.

Many companies are developing integrated systems for bacterial identification and AST (Neely Lori et al., 2013; Li et al., 2017; Gite et al., 2018; Pancholi et al., 2018). For example, MALDI-TOF mass spectrometry (MALDI-TOF MS) and Vitek-2 or BD Phoenix have been commonly used in the clinic for identification and AST, respectively (Yang et al., 2018; van Belkum et al., 2019; Weis et al., 2020). However, these systems require isolated bacteria and are based on measuring bacterial growth and turbidity changes, which are slow and generally take 2–3 days. Accelerated diagnosis developed an automatic digital microscope system, which integrates fluorescence *in situ* hybridization (FISH) and morphokinetic cellular analysis for identification and AST (90 min for identification, ~7 h for AST from positive blood culture bottle; Pancholi et al., 2018). However, FISH requires one or several specific probes and cannot reach 100% hybridization, therefore is easy to lose signals, resulting in false-negative results (Schimak et al., 2016). T2 Biosystems and First Light Biosciences are also developing rapid bacterial identification and AST using magnetic nanoparticles and antibodies (Neely Lori et al., 2013; Gite et al., 2018). However, they have not been widely implemented because they have not been extended to different pathogens. An ideal identification and AST system should be universal to all bacteria, use less or no additional markers, and obtain identification and AST of bacteria in one system to reduce manual operation, turnover processes, and contamination.

Raman-based technology is an emerging approach for bacterial identification by measuring the spectral differences in bacteria and AST by monitoring bacteria’s spectral response to antibiotic treatment (Dina et al., 2017; Ivleva et al., 2017; Fang et al., 2019; Ho et al., 2019; Thrift et al., 2020; Bashir et al., 2021). Especially, Raman-based technology using bacterial metabolism as a marker has proven to be a promising alternative for rapid AST (Tao et al., 2017; Hong et al., 2018; Yang et al., 2019; Bauer et al., 2020; Yi et al., 2021). In this approach, bacterial metabolism was selectively probed *via* monitoring the conversion of D_2_O or deuterated glucose into biomolecules (Hong et al., 2018; Wang et al., 2020). However, drug susceptibility detection using spontaneous Raman requires a large amount of spectral data and a long integration time, limiting its application in rapid AST. To increase the speed and throughput of AST, stimulated Raman scattering (SRS) microscopy has been used to measure the *de novo* synthesis of C−D bonds within single bacteria for rapid AST (Hong et al., 2018; Zhang et al., 2020, 2021). It is demonstrated that single-cell metabolism inactivation concentration (SC-MIC) can be obtained within 2 h using SRS microscopy (Zhang et al., 2020). In addition, Raman spectroscopy, with its non-destructive and chemical selectivity characteristics, has also been applied in microbial identification (Ho et al., 2019; Liu et al., 2020; Wang et al., 2021; Yan et al., 2021). Because of the heterogeneity of microorganisms, it is crucial to collect many phenotypes of different individual bacteria in the same population (Heyse et al., 2019). As a result, convolutional neural networks (CNN) that can efficiently process large amounts of data demonstrate extraordinary advantages in bacterial identification. Nevertheless, rapid identification and AST with Raman technology have not been demonstrated in one system.

Here, we demonstrate a compound Raman microscopy (CRM) system, which integrates Raman spectroscopy and SRS microscopy in one system, for rapid bacterial identification and AST. The CRM takes advantage of chemical selectivity in Raman spectroscopy for bacterial identification while utilizing the high-throughput nature of SRS imaging for AST. We first generated an extensive bacterial Raman spectral dataset and applied deep learning to identify six common bacterial pathogens. Then, bacterial metabolic activity was quantitated by SRS to determine the SC-MIC for AST. Using *S. aureus* and *E. coli* as models, we demonstrated the ability of this CRM system to achieve bacterial Gram-staining classification within 0.5 h and AST within 2.5 h directly from urine samples.

## Materials and methods

### Bacteria and antibiotics

Bacterial strains used in this study (Supplementary Table S1) were purchased from the Beijing Microbiological Culture Collection Center or provided by the Institute of Clinical Pharmacology, Peking University. All bacterial strains were mixed with 2.5% glycerol and frozen in a cryopreserved tube (Jinzhang, Tianjin, China) at −80°C for storage. For experiments, the frozen strain was thawed and cultured on agar plates with Luria broth (LB) medium (Sigma Aldrich) at 35°C for 24 h before use. Antibiotic solution (gentamicin) was filtered through a 0.22-μm sterile syringe filter (Millipore Millex, Burlington, MA) and stored at −80°C before use.

### Preparation of bacterial samples

A D_2_O medium was prepared by adding LB powder to a D_2_O solution at a final concentration of 2.5% in weight. The preparation of bacteria samples is similar to our previous study (Zhang et al., 2021). In brief, bacteria were diluted in LB media to a 0.5 McFarland standard. Then, the bacterial solution was further diluted in D_2_O media to ~6 × 10^5^ CFU mL^−1^. After incubation for a period of time, about 500 μl sample was centrifuged and fixed with 10% formalin solution. Next, about 3 μl bacterial solution was deposited on a polylysine-coated coverglass; then, another non-coated coverglass was placed on top of the polylysine-coated coverglass for SRS imaging. For the urine samples, we first filtered the samples with a 5 μm filter to remove large debris in the urine and then followed the same procedure as above.

### CRM system and data processing

The CRM system is based on a picosecond laser system (picoEMERALD, Applied Physics & Electronics), which outputs synchronized pump and Stokes beams. The wavelength of the pump beam is tunable from 700 to 990 nm, with a pulse width of 2 ps and a repetition rate of 80 MHz. The wavelength of the Stokes beam is fixed at 1,031 nm, with a pulse width of 2 ps and a repetition rate of 80 MHz. The Stokes beam was modulated at 20 MHz by an electro-optical modulator. For SRS imaging at C–D vibrations, the pump beam was tuned to 842 nm. Each image contains 200 × 200 pixels with a pixel dwell time of 50 μs. When performing Raman spectroscopy measurement, the pump beam, used as the excitation laser, was tuned to 707 nm. Raman signal from the sample was detected by a spectrometer (KYMERA-328I-A, Andor). A CCD camera was used to determine the location of bacteria. The laser power at the sample was ~10 mW after a 60× water objective (Olympus MPLAN), the acquisition time was 1 s, and the grating was set at 300 L/mm.

The original Raman spectra contain noise and background, and therefore, the spectra need to be processed before deep learning. The pre-processing takes four steps: (1) wavenumber selection; (2) background subtraction; (3) smoothing; (4) normalization. In brief, the wavenumber between 400 and 1,800 cm^−1^ was selected as the region of interest. An asymmetric least-squares method was applied to subtract the background signal. Then a Savitzky–Golay filter smoothed the data to reduce the noise. All the processing mentioned above was done by *Python 3.7 scipy 1.2.1*.

### CNN architecture

A CNN based on AlexNet was used to classify bacteria (Erzina et al., 2020). As the input of the CNN model, the pre-processed spectra were first connected to two convolutional layers, which contained 8 and 16 kernels, respectively. Each layer was followed by a batch normalization layer and a max-pooling layer with a pool size set at 2. The data were then concatenated to 1 dimension and input into a fully connected layer with 3 layers of neurons, containing 64, 32, and 6 neurons. The 6 neurons represented the output probability corresponding to 6 different types of bacteria.

After two fully connected layers, the output layer of the network was connected, corresponding to the final classification results. The number of neurons in the output layer was the number of data categories. For the six-classification network in bacterial identification, the number of neurons in the output layer was 6. The SoftMax function was used to achieve multi-classification. The total output value is 1, and the corresponding predicted probabilities of *A*. *baumannii*, *E*. *coli*, *E*. *faecium*, *K*. *pneumoniae, P*. *aeruginosa*, and *S*. *aureus* were the output of the trained networks. This CNN model was based on *Python 3.7 tensorflow 1.14.0* and *keras 2.2.4*.

### Training and evaluation

The dataset was split into a training set and a test set in a ratio of 7:3. The CNN model was first trained using the data from the training set, during which the validation size, batch size, dropout rate, and epoch were set as 30%, 60, 0.6, and 100, respectively. For each epoch, the training set contained 354 spectra, while the validation set contained 150 spectra.

Since the dataset was not large enough to create a test set that could provide convincing results, data augmentation was applied during the testing process. By shuffling the spectra, we created a lot more data with a slight difference that significantly increased the size of the test set while making the testing outcomes more convincing. In this study, the augmentation was set to be 100 times and rehabilitated during the testing process.

### Broth microdilution (BMD) test

The BMD test was used as a reference recommended by the Clinical and Laboratory Standards Institute (CLSI; Clinical and Laboratory Standards Institute (CLSI). Methods for Dilution Antimicrobial Susceptibility Tests for Bacteria That Grow Aerobically. 11th ed. CLSI standard M07. Wayne, PA: CLSI; 2018). In brief, bacteria were cultured in Mueller Hinton Broth (MHB) media (Sigma Aldrich) in 96-well plates. Antibiotic solution, using triplicate samples, was added to the plate with two-fold serial dilution. A bacterial stock solution was pipetted into the 96-well plates at a final concentration of 5 × 10^5^ CFU mL^−1^. The MIC values were determined after incubation at 35°C for 16–20 h.

## Results

### Performance of the CRM

**Figure 1:**
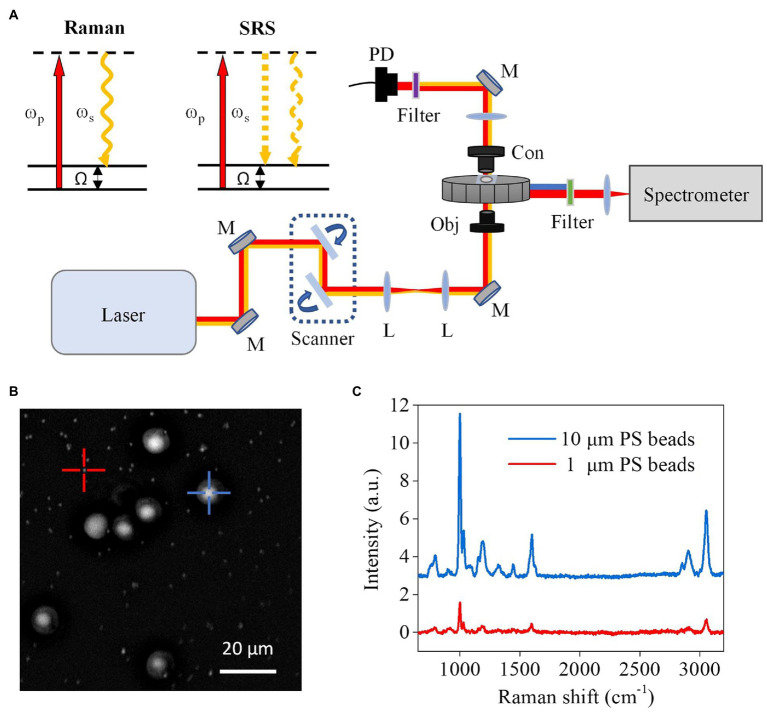
Schematic setup and performance of the CRM system. **(A)** Schematic setup and diagrams of spontaneous Raman and SRS microscopy. ω_p_, pump beam; ω_s_, Stokes beam; Ω, Raman-active molecular vibration; L, lens; M, mirror; Obj, objective; Con, condenser; PD, photodiode. **(B)** SRS image of a mixture of PS beads with 1 μm and 10 μm diameters. **(C)** Raman spectra obtained from 1 μm and 10 μm beads in **(B)**, respectively.

We used polystyrene (PS) beads of known diameters to evaluate the performance of our CRM setup (Figure 1A). Using the forward-detected SRS, single PS beads with diameters of 1 μm and 10 μm distributed on a coverglass were observed (Figure 1B). Meanwhile, Raman spectra of the beads were obtained (Figure 1C). The ring breathing band at 1,005 cm^−1^ indicates the correct measurement of the PS beads. These results demonstrate the ability of our CRM to obtain both SRS images and Raman spectra of a point of interest.

### Workflow of rapid identification and AST of bacteria with the CRM

**Figure 2:**
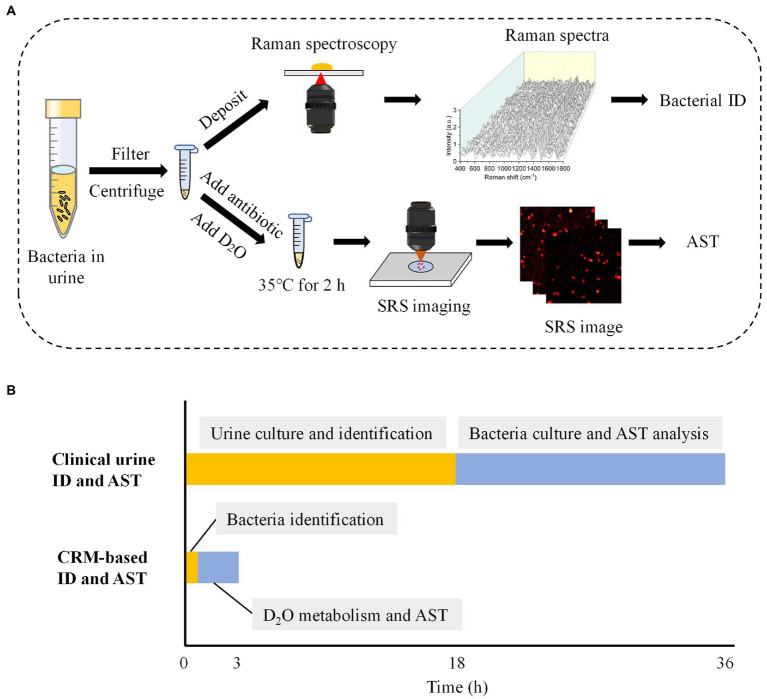
Workflow of the CRM for rapid identification and AST in urine. **(A)** The flow chart of bacterial identification and AST for urine samples. **(B)** Turnaround time required for the conventional and CRM-based bacterial identification and AST for urine samples. The time estimation was based on optimal conditions. ID, identification.

Rapid identification and AST of pathogenic bacteria with the CRM have two steps (Figure 2A). In the first step, we concentrated urine samples by filtration and centrifugation. Then, Raman spectra of the samples were obtained and compared with Raman databases we established to obtain bacterial identification results. This step, including sample processing and Raman spectra measurement, takes ~0.5 h (Figure 2B).

In the second step, we first treated bacteria with a selected antibiotic-containing medium for 1 h. Then, D_2_O mediums containing different concentrations of antibiotics were added to the bacteria solution for an additional 1 h. Notably, the antibiotic concentration was kept the same as the initial concentration used in each sample, meanwhile reaching a final D_2_O concentration of 70%. This D_2_O concentration was previously demonstrated to cause negligible toxicity to most bacteria species (Zhang et al., 2020). With SRS imaging and automatic data processing (Supplementary Figure S1), the SC-MIC was next determined to obtain the AST results. In total, bacterial identification and AST with our CRM system take ~3 h from a urine sample. Compared with the current UTI diagnostic gold standard, which takes approximately 36 h, our CRM significantly improved the speed of bacterial identification and AST (Figure 2B).

### Establishment of a deep learning model for bacterial identification

**Figure 3:**
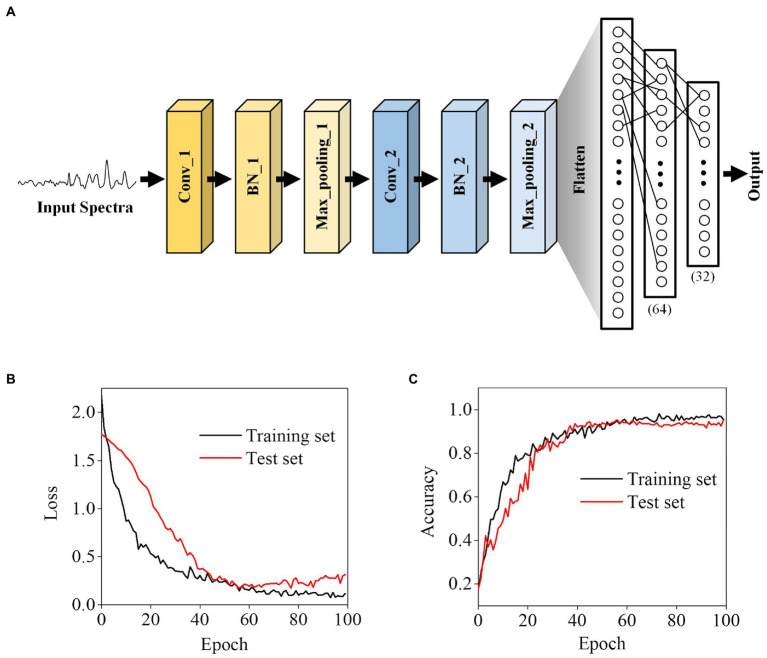
Deep learning-based bacterial classification. **(A)** The architecture of the Alexnet-based deep learning model. **(B)** Training loss and **(C)** accuracy. The displayed loss and accuracy were the values obtained with the dropout layer deactivated after training at each epoch.

To identify bacteria with Raman spectroscopy, we developed an Alexnet-based deep learning model (Figure 3A). Instead of the traditional rule, our model uses the linear activation function when building the convolutional network, which is used to save all the convolutional information, including positive and negative correlations. The Leaky Relu layer and the Dropout layer were used in the network to avoid over-fitting.

In neural networks, over-fitting corresponding to specific data is one of the main problems. To avoid over-fitting, we split the data into a training set, which was used to train the model, and test sets in a ratio of 7:3 to verify the model. Through learning iterations, there is no significant difference between the loss and accuracy of the test and training sets, indicating that the optimized model did not have the over-fitting problems (Figures 3B,C).

### Performance of the deep learning model in bacterial identification

**Figure 4:**
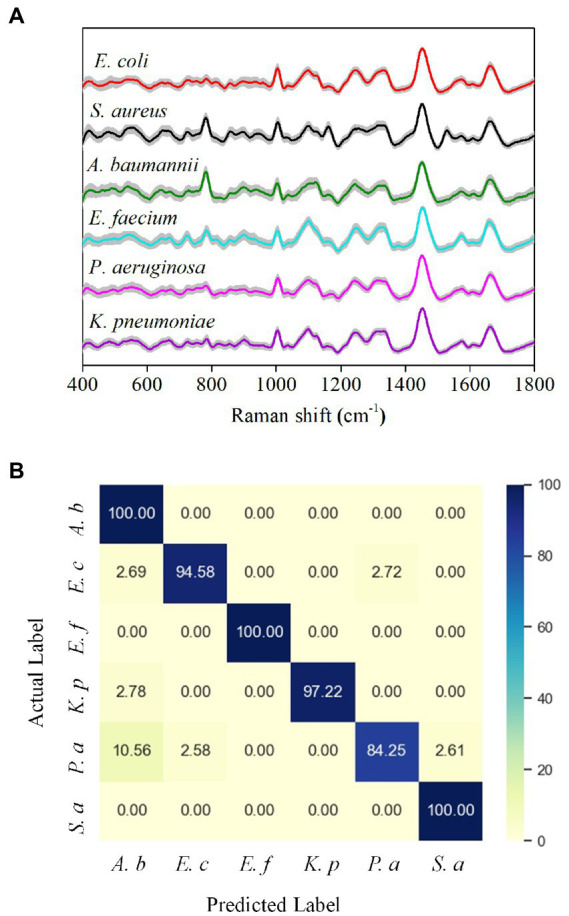
Performance of the Alexnet-based deep learning model in bacterial identification. **(A)** Average Raman spectra of six common bacteria in UTI. The gray area represents the standard deviation of the spectra. **(B)** Confusion matrix of bacterial classification (values are listed as percentages).

We next trained a neural network on a 6-class isolate recognition task. CNN outputs a probability distribution across 6 reference isolates and uses the maximum value as the predicted class. The model was trained on a reference data set and then tested on an independent test data set. To minimize the deviation caused by cell heterogeneity and physiological status of the same strains, we obtained 120 Raman spectra from each bacteria species to avoid potential differentiation caused by cellular frameworks. Our spectral data ranged from 400 to 1,800 cm^−1^ and consisted of 2,000 one-dimensional float data. Raman spectra of different bacterial media, D_2_O, D_2_O LB, and water, showed that bacterial Raman spectra were not affected by these media (Supplementary Figure S2). Figure 4A shows the average Raman spectrum (color line) of the pathogens tested, where the intraspecies variation of each strain appears as a gray area around each spectrum. The Raman spectra show high similarity between different species, probably due to their similar biochemical components. Nevertheless, it is difficult to differentiate the species with naked eyes only.

We next used the CNN model to classify bacteria based on the differences in Raman spectra. As shown in the confusion matrix, the six-class model achieved an average classification accuracy of 96.0% (Figure 4B). Only *P*. *aeruginosa* has a recognition sensitivity below 90%, while the other bacterial species all have a recognition sensitivity of over 90%. The classification specificities are between 97% and 100% (Supplementary Table S1). Furthermore, we also compared our deep learning model with a principal component analysis (PCA) model, an unsupervised multivariate analysis tool for spectral data management (Dina et al., 2017; Yan et al., 2021) for identification. Due to the minor inter-class differences in the bacterial Raman spectrum data set and the relatively significant intra-class differences, traditional machine learning algorithms, such as linear discriminant analysis (LDA), support vector machines (SVM), and logical regression, are often difficult to achieve an ideal classification effect (Ho et al., 2019; Yan et al., 2021). We used PCA-LDA and a cross-validation method to test the classification performance (Supplementary Figures S3A,B). The PCA-LDA results show that the sensitivity, specificity, and accuracy after averaging are 56.5, 91.3, and 85.5%, respectively (Supplementary Table S2), and therefore demonstrate that the classification effect of PCA-LDA is not ideal. Collectively, these results show that the deep learning model has significantly better indicators than the PCA-LDA model for bacterial identification.

### Rapid identification and AST in urine with the CRM

**Figure 5:**
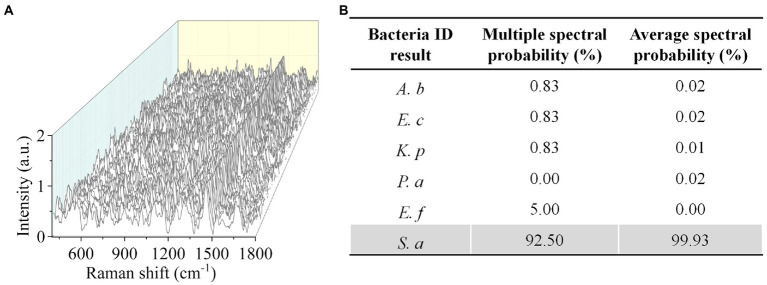
Rapid identification of *S. aureus* in urine by CRM. **(A)** Raman spectra of *S. aureus*. **(B)** Identification results by the deep learning model. *A. baumannii* (*A. b***)**, *E. coli* (*E. c***)**, *E. faecium* (*E. f*), *K. pneumoniae* (*K. p*), *P. aeruginosa* (*P. a*), and *S. aureus* (*S. a*).

**Figure 6:**
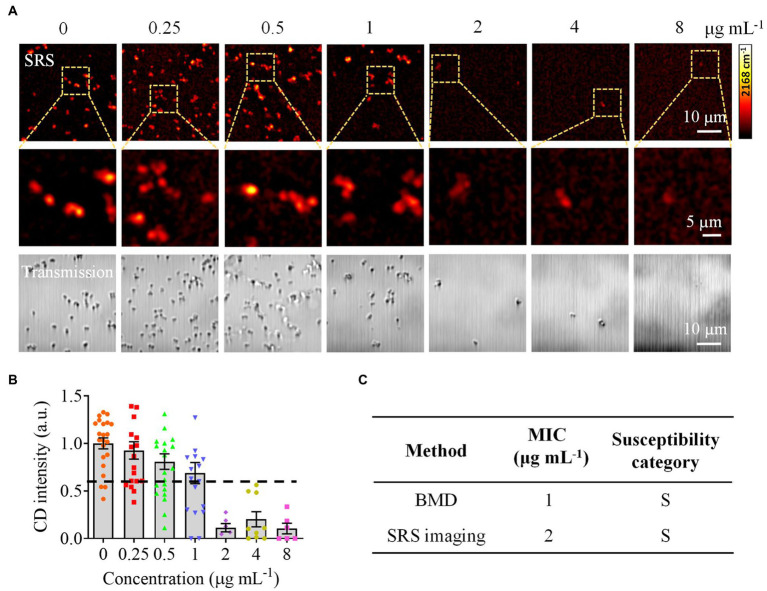
Rapid AST of *S*. *aureus* in urine by CRM. **(A)** SRS at C–D and corresponding transmission images of *S*. *aureus* (MIC_BMD_ = 1 μg mL^−1^) after culture in a D_2_O-containing medium with serially diluted gentamicin. **(B)** Statistical analysis of the C–D intensity in *S. aureus* in **(A)**. **(C)** Comparison of the MIC and susceptibility category determined by the BMD and SRS imaging-based methods. S, sensitive. Error bars represent the standard error of the mean (SEM).

To validate the effectiveness of CRM in clinically relevant scenarios, we next tested bacteria in urine samples. We used spiked samples to mimic the clinical UTI samples by adding bacteria to urine at a final concentration of ~10^6^ CFU mL^−1^. We first tested *S*. *aureus* and obtained 120 Raman spectra from different sample locations (Figure 5A). By comparing the spectra with the established data set, we correctly identified *S*. *aureus* with a multiple spectral probability of 92.5% and an average spectral probability of 99.9% (Figure 5B). Here, to calculate the multiple spectral probability, we compared each spectrum to the established data set to predict an output label. Then the multiple spectral probability was defined as the probability corresponding to each label, i.e., the proportion of the number of data predicted in this category to the total amount of data. To calculate the average spectral probability, we first averaged all the spectra obtained. Then the average spectral probability was defined as the output probability of each label predicted from this averaged spectrum. Once the identification result was obtained, we next performed AST with SRS. We tested *S*. *aureus* toward gentamicin (Figure 6A). Using the same threshold established in our previous work (Zhang et al., 2020), the statistical analysis of bacterial C–D signals determined the SC-MIC to be 2 μg mL^−1^ (Figure 6B). This result has the same essential and category agreement as the MIC result obtained from the BMD method (Figure 6C).

Following the same procedure as *S*. *aureus*, we further tested *E. coli*, which is also commonly encountered in UTI. Although the identification of *E. coli* is not ideal at the species level, the Gram-staining classification achieved an accuracy of 100% by analyzing the Raman spectral multiple values (Supplementary Figure S4A). Meanwhile, SRS imaging (Supplementary Figure S4B) and statistical analysis (Supplementary Figure S4C) determined the SC-MIC to be 1 μg mL^−1^, which has the same essential and category agreement as the BMD result (Supplementary Figure S4D).

Furthermore, to assess the feasibility of CRM for UTI in a clinical setting, we tested six clinical strains, including three *E*. *faecium* and three *E*. *coli*. Our results showed that all the clinical *E. faecium* strains were successfully identified, with an average spectral probability of 97.2% (Supplementary Tables S3A–C). Meanwhile, the Gram-stain classification for the clinical *E*. *coli* strains achieved an average accuracy of 94.2% (Supplementary Tables S4A–C). In addition, we conducted a direct blind test of five clinical urine samples by the CRM. Although the identification results were not ideal, the average accuracy of the Gram-staining classification exceeded 96% (Supplementary Tables S5A–E). Considering that two strains in the samples were not even in the six species in which the model was built, our Gram-staining classification results were satisfactory. Since the selection of antibiotics tested in AST is dependent on the Gram-staining feature, pre-identifying the Gram-staining characteristics of pathogens could also significantly accelerate the turnaround time of AST (Yi et al., 2021). These results collectively proved that our CRM is suitable for clinical application to urinary pathogens.

## Discussion and conclusion

For bacterial identification, current culture-free methods include fluorescence (Park et al., 2019; Yi et al., 2020), FISH (Phung et al., 2017), and magnetic nanoparticles (Neely Lori et al., 2013). Compared with these techniques, Raman spectroscopy does not require specially designed markers that are needed in these methods. In addition, an integrated system based on droplet microfluidic-driven single-cell diagnostics offers great promise in rapid microbial identification and susceptibility detection (Hsieh et al., 2022). Although this system reduces the diagnostic time of UTIs, additional markers are still needed.

Cheng et al. used a compound Raman to perform high-speed vibration imaging and spectral analysis of liposomes (Slipchenko et al., 2009). In their setup, Raman spectroscopy was integrated with coherent anti-Stokes Raman scattering (CARS) microscopy, another imaging modality of coherent Raman scattering microscopy. Compared with CARS, SRS does not suffer from the non-resonant background that dramatically reduces imaging contrast (Cheng and Xie, 2015). In the future, to cope with the complicated clinical needs, the design of cardboard to minimize manual sample preparation, autofocusing, and auto measurement of the sample are essential for the standardization of the CRM.

In summary, we demonstrated an integrated CRM, which includes Raman spectroscopy and SRS imaging, for rapid identification and AST of bacteria in urine. To achieve this, we generated a Raman spectral dataset and developed a deep learning model to identify six common bacterial pathogens in UTI. In addition, we applied SRS to quantify bacterial metabolism to determine their antimicrobial susceptibility. Importantly, our CRM system can achieve Gram-staining classification and provide phenotypic AST results within 3 h directly from urine samples, a significant time-saving compared to the conventional methods.

## Data availability statement

The original contributions presented in the study are included in the article/Supplementary material, further inquiries can be directed to the corresponding authors.

## Author contributions

WZ, WH, LY, LZ, and PW conceived and designed the project. WZ, SH, and YW prepared the culture and samples. HS and XC carried out the algorithm design. WZ and SH performed the experiments. WZ, WH, and PW wrote the manuscript. All authors contributed to the article and approved the submitted version.

## Funding

This work was supported by the National Natural Science Foundation of China (81901790) and the Key R&D program of the Ministry of Science and Technology (2020YFC2005405).

## Conflict of interest

The authors declare that the research was conducted in the absence of any commercial or financial relationships that could be construed as a potential conflict of interest.

## Publisher’s note

All claims expressed in this article are solely those of the authors and do not necessarily represent those of their affiliated organizations, or those of the publisher, the editors and the reviewers. Any product that may be evaluated in this article, or claim that may be made by its manufacturer, is not guaranteed or endorsed by the publisher.
