# Supplementary material for: Compound Raman microscopy for rapid diagnosis and antimicrobial susceptibility testing of pathogenic bacteria in urine
Source: Front Microbiol. 2022 Aug 24;13:874966. doi: 10.3389/fmicb.2022.874966 (PMC9449455; doi:10.3389/fmicb.2022.874966)
Supplement: Supplementary file 1 [file Table_1.DOCX]

Supplementary Material

## Supplemental Table S1

| **Bacteria** | **Sensitivity (%)** | **Specificity (%)** | **Accuracy (%)** |
| --- | --- | --- | --- |
| *A. b* | 100 | 97.4 | 97.8 |
| *E. c* | 94.6 | 99.5 | 98.7 |
| *E. f* | 100 | 100 | 100 |
| *K. p* | 97.2 | 100 | 99.5 |
| *P. a* | 84.3 | 99.5 | 96.9 |
| *S. a* | 100 | 99.5 | 99.6 |
| **Mean** | 96.0 | 99.3 | 98.8 |

**Evaluation of deep learning-based six bacteria classification.** *A. baumannii* (*A. b*), *E. coli* (*E. c*), *E. faecium* (*E. f*), *K. pneumoniae* (*K. p*)*, P. aeruginosa* (*P. a*) and *S. aureus* (*S. a*).

## Supplemental Figure S1

##
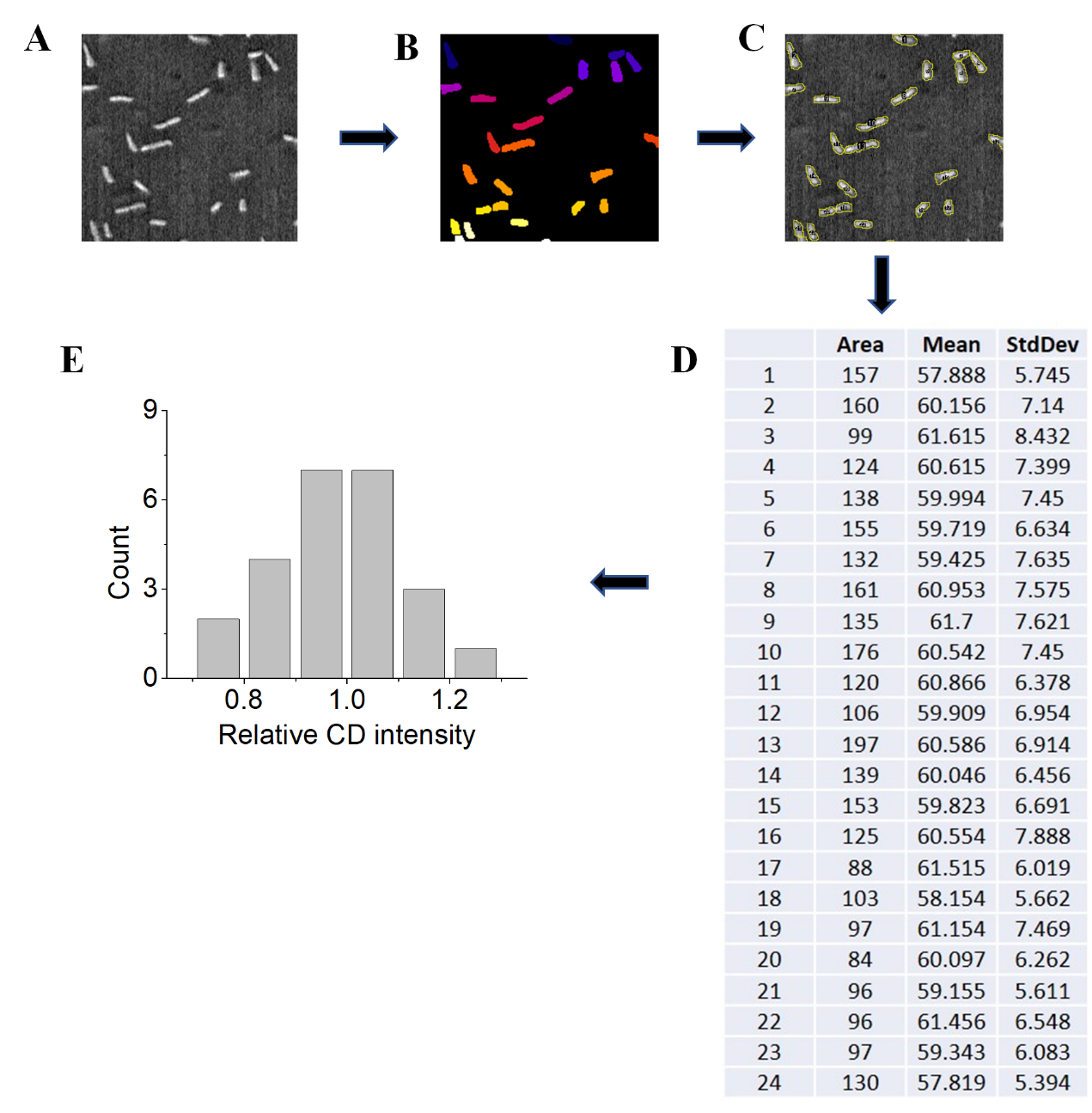


**Automated image processing algorithm was implemented by ImageJ.** (A) Original image. (B) Image after threshold adjustment to determine the area of bacterial and particle analysis. (C) The corresponding data points selected in the raw image. (D) Results of the corresponding data points in the raw image. (E) Statistical results of the average C–D intensity of the data points after subtraction of background.

## Supplemental Figure S2

##
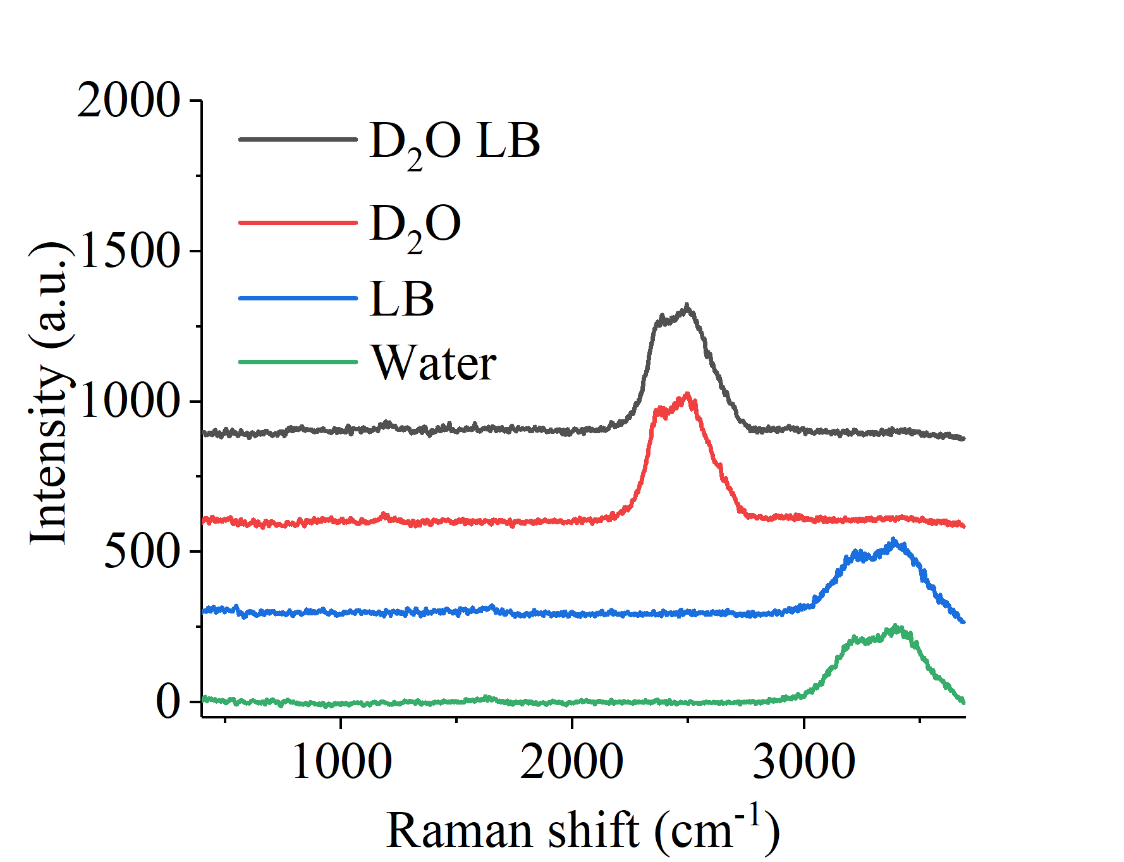


**Spontaneous Raman spectra of D_2_O LB, D_2_O, LB and Water.**

## Supplemental Figure S3

##
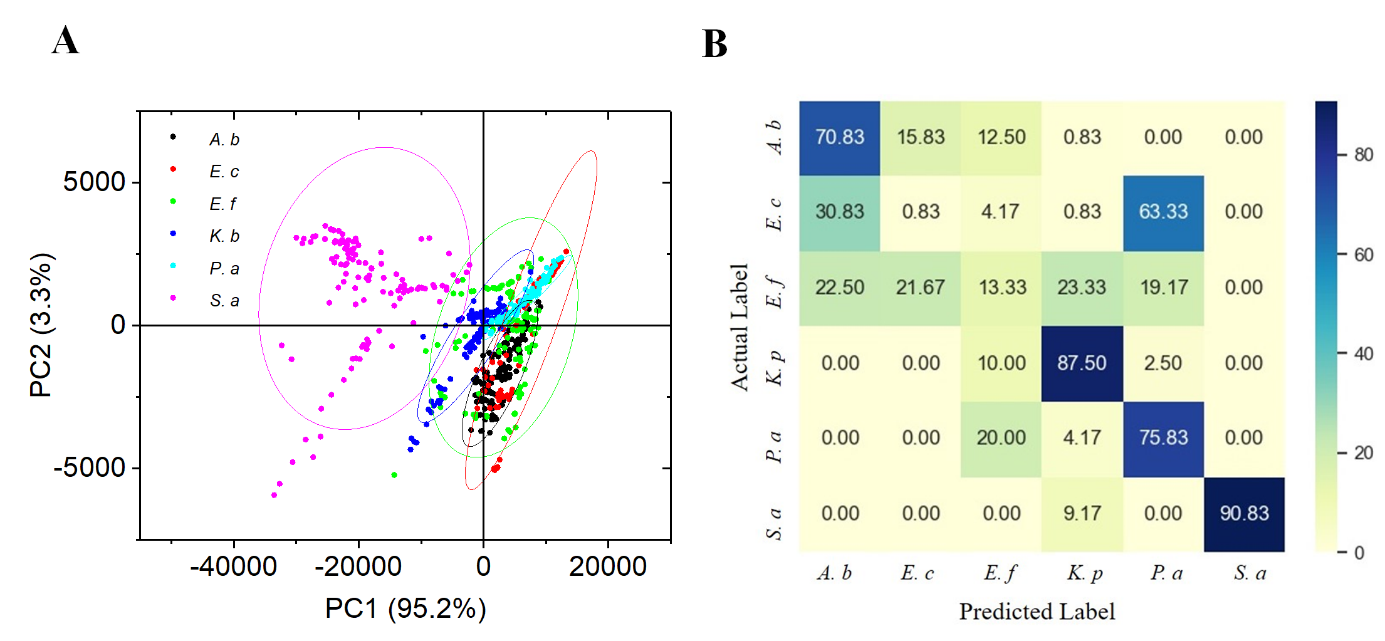


**Discrimination performance of PCA-LDA.** The PCA plots of (A) and confusion matrix (B) for six strain classes (values are listed as percentages).

## Supplemental Table S2

| **Model** | **Sensitivity (%)** | **Specificity (%)** | **Precision (%)** |
| --- | --- | --- | --- |
| PCA-LDA | 56.5 | 91.3 | 85.5 |
| CNN | 96.0 | 99.3 | 98.8 |

**Evaluation of PCA-LDA and CNN classification models.**

## Supplemental Figure S4

##
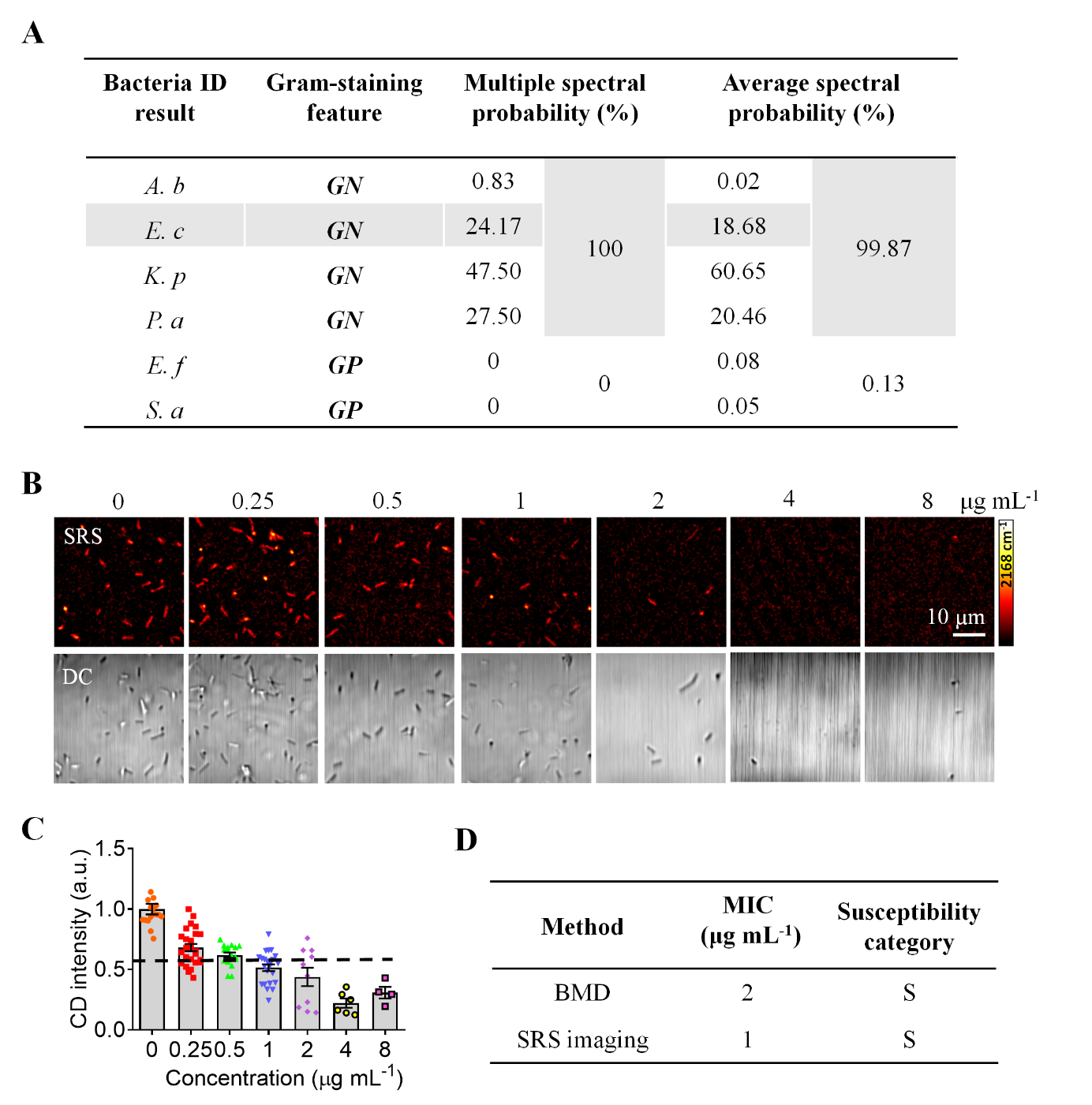


**Rapid Identification and AST of *E. coli* by compound Raman microscope.** (A) Bacteria Identification result by the Alexnet-based deep learning model. GN: Gram-negative. GP: Gram-positive. (B) SRS at C–D and corresponding transmission images of *E. coli* (MIC_BMD_= 2 μg mL^-1^) after culture in D_2_O containing medium with the addition of serially diluted gentamicin. Scale bar: 10 µm. (C) Statistical analysis of C–D intensity in *E. coli* in (B). (D) Comparison of the MICs determined by the BMD method and the SRS imaging method with the CLSI susceptibility category. S: sensitive. Error bars represent the standard error of the mean (SEM).

## Supplemental Table S3


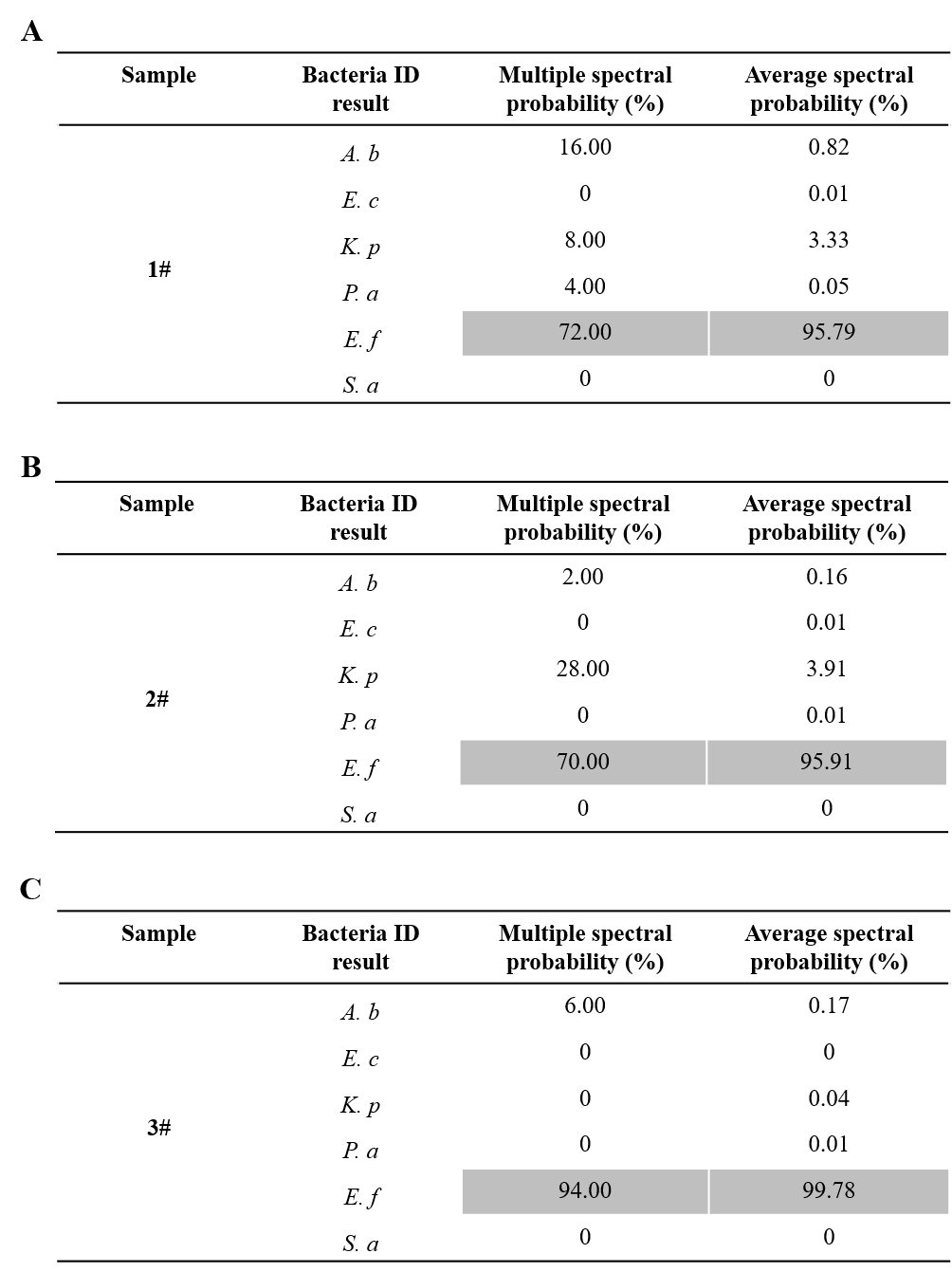


**The identification results of three clinical isolates (*E. faecalis*) by deep learning model.**

## Supplemental Table S4


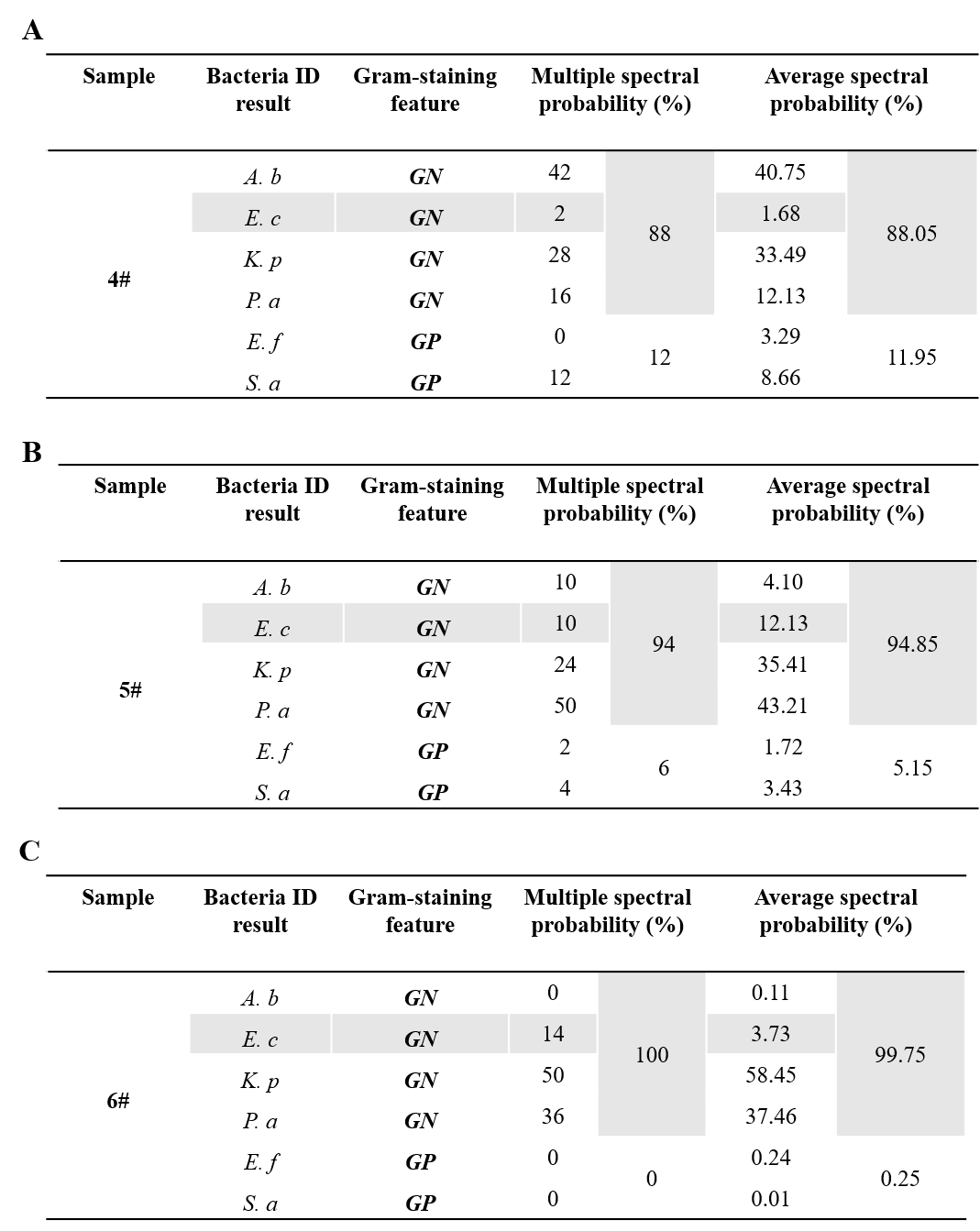


**The identification results of three clinical isolates (*E. coli*) by deep learning model.** The clinical strains (A-C) were from the Institute of Clinical Pharmacology, Peking University.

## Supplemental Table S5


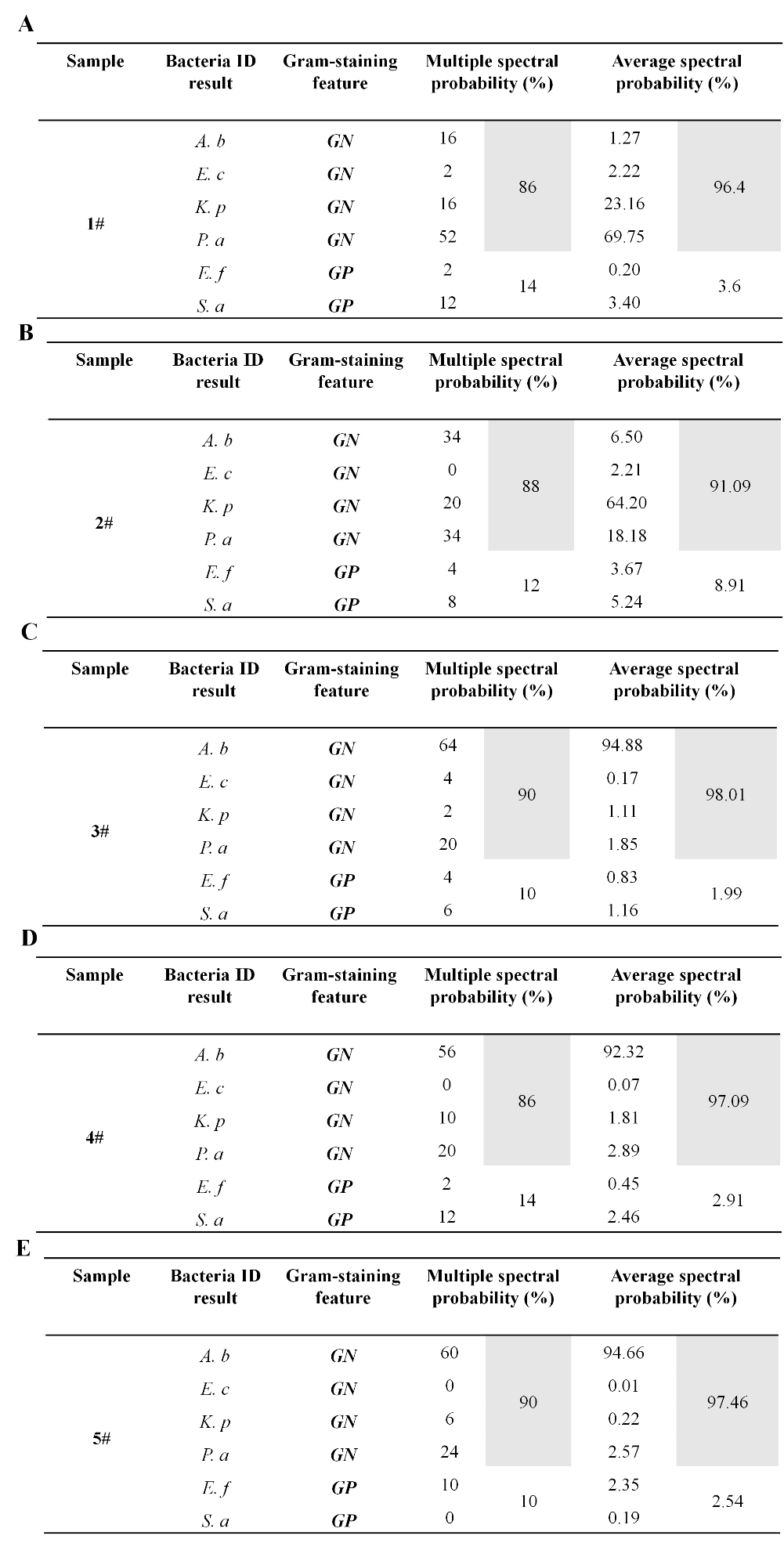


**The identification results of five clinical urine samples by deep learning model.** The clinical urine samples (1# was *E. coli*, 2# was *E. coli*, 3# was *E. coli*, 4# was a mixture of *S. marcescens* and *P. mirabilis,* 5# was *C. freundii*) were both from the Department of Clinical Laboratory, Beijing Boai Hospital, China Rehabilitation Research Center.
